# Genetic characterization of Markhoz goat breed using microsatellite markers

**DOI:** 10.5194/aab-61-469-2018

**Published:** 2018-12-06

**Authors:** Fariborz Asroush, Seyed-Ziaeddin Mirhoseini, Nejat Badbarin, Alireza Seidavi, Vincenzo Tufarelli, Vito Laudadio, Cataldo Dario, Maria Selvaggi

**Affiliations:** 1 Department of Animal Science, Rasht Branch, Islamic Azad University, Rasht, Iran; 2 Department of Animal Science, University of Guilan, Rasht, Iran; 3 Department DETO – Section of Veterinary Science and Animal Production, University of Bari, Valenzano, Italy; 4 Department of Agro-Environmental and Territorial Sciences, University of Bari, Bari, Italy

## Abstract

The use of molecular markers can support the management of endangered populations and should be combined with appropriate breeding strategies to improve productive traits avoiding the decline of the breed. The genetic variability at 10 microsatellite loci were investigated in a sample of 100 unrelated Markhoz goats (77 females and 23 males). The investigated population was reared at the Sanandaj Markhoz goat Performance Testing Station in Kurdistan, Iran. Markhoz goat, a multipurpose breed, is one of the most valuable genetic resources in Iran. All the studied loci were found to be polymorphic and a total number of 52 alleles were identified with an average number of alleles of 5.2. Moreover, some population genetic indices, such as observed and expected heterozygosity, observed and expected number of alleles, Shannon's index, Nei's expected heterozygosity, and polymorphism information content were also calculated. Despite the decreasing population size, Markhoz goat genetic diversity is still conserved. The breed seems to have a good level of genetic variability and, as a consequence, a potential margin of adaptability to environment and for future genetic improvement.

## 1 Introduction

Goat farming is a vital part of the national economy in many countries, especially in the Mediterranean and Middle Eastern regions. Goats are able to provide high-quality products under diverse climatic conditions, being resilient to extreme and capricious environments (Selvaggi and Tufarelli, 2012; Selvaggi et al., 2014a). Moreover, local goat breeds play a significant role in the sociocultural life of the rural community and in preserving marginal areas. (Selvaggi et al., 2014a, b).

Undoubtedly, the effective management of genetic resources of farm animals requires comprehensive knowledge about breed characteristics, data on the size and structure of the populations, and the geographical distribution of genetic diversity within and between breeds (Groeneveld et al., 2010).

Markhoz goat breed is one of the most valuable genetic resources in Iran. This breed is well adapted to the environmental conditions of arid and semi-arid areas where it is used as a multipurpose animal for the production of milk, meat, hair and hide (Farshad et al., 2008). Markhoz goat has a coat called mohair being the only mohair goat breed in Kurdistan province where it represents the main economic income for goat producers.

Twenty years ago, about 25 000 Markhoz goats were raised in Kurdistan province of Iran. Latest census data reported a total population size of 1669 does and bucks (Bahmani et al., 2015). This rapid number decrease is due to many different reasons such as discouragement in youth and family members, shortage of shepherds, nutrition problems, existence of haired goat breeds with higher income, low income of goat raising and low financial feasibility for herders. Although Markhoz goat is not at risk of extinction, when considering the downward trend, this breed may face the risk of genetic erosion due to natural disasters and epidemic diseases (Bahmani et al., 2011).

Therefore, given the importance of this breed and the need to identify genetic variability in the Markhoz goat population, it is necessary to assess the genetic diversity within this breed. In fact, genetic diversity is fundamental for long-term survival of populations providing the raw material for adaptation and evolution, especially when environmental conditions have changed (Eriksson et al., 1993). Thus, the genetic characterization of native breeds is the first step for their conservation. The use of highly variable molecular genetic markers, such as microsatellites, is one of the most powerful tools to study genetic diversity because of its high polymorphic degree, random distribution across genome and neutrality with respect to selection (Kemp et al., 1995). Therefore, the present study aimed to genetically characterize the Markhoz goat breed using specific microsatellite markers to investigate the genetic diversity within this population and to improve future breeding strategies and conservation programmes.

## 2 Materials and methods

Individual blood samples (10 mL each) were collected in K3-EDTA tubes and stored at -25 ${}^{\circ }$C from 100 unrelated goats (77 females and 23 males) belonging to Markhoz breed reared at the Sanandaj Markhoz goat Performance Testing Station in Kurdistan province of Iran. Considering the small population size, the selection of unrelated animals to be included in this study did not allow us to extend this investigation to a larger number of individuals. Genomic DNA was isolated from whole blood using a ZR Genomic DNA II Kit (Zymo Research, Irvine, CA, USA). The purity and concentration of DNA was measured spectrophotometrically by calculating the ratio of optical densities at 260 and 280 nm.

Ten microsatellite markers, previously described as polymorphic in goat, were investigated: BM1312, LSCV06, ILSTS082, IDVGA64, LSCV25, BMS1248, BM2830, IL2RA, LSCV36 and MAP2. The studied microsatellites are located on chromosomes 1, 2, 5, 13 and 19 (Table 1).

**Table 1:** Primer characteristics of amplified microsatellite loci.

Chromosome number	Marker	Primers	Type sequence
1	BM1312	CCATGTGCTGCAACTATGAC GGAATGTTACTGAACCTCTCCG	Forward Reverse
1	LSCV06	GACTTCTCCCAGTAGGCTGG GCTGTTCGGAAGTGATGTAG	Forward Reverse
2	ILSTS082	GCTGTTCGGAAGTGATGTAG AGAGGATTACACCAATCACC	Forward Reverse
2	IDVGA64	GCAGAGGAGGTTTTCAGATTC CGGAGATCAGAGCACTTGTC	Forward Reverse
5	LSCV25	CTCCTTACCATCCCGATTAG GAGGTGTCTGAGGGAAGAG	Forward Reverse
5	BMS1248	GTAATGTAGCCTTTTGTGCCG TCACCAACATGAGATAGTGTGC	Forward Reverse
5	BM2830	AATGGGCGTATAAACACAGATG TGAGTCCTGTCACCATCAGC	Forward Reverse
13	IL2RA	AGCAGAGGTACAGGTGGTAAGCA GATATGCCTTGGAGAAGGTAGCGTAT	Forward Reverse
19	LSCV36	GAGTTTTCAGAGCCTTCAGC CTTATGTGTGTTTGCTCACAG	Forward Reverse
19	MAP2	AAGGATTCTGTCTGATACCACTTAG TTTACCAGACAGTTTAGTTTTGAGC	Forward Reverse

PCR amplifications were carried out in a 25 µL total volume containing 50 ng DNA template, 1× PCR buffer, 5 µM of each primer, 250 µM dNTP Mix, 1.5 mM ${\mathrm{MgCl}}_{2}$ and 1 unit of Taq DNA polymerase. The microsatellites were amplified with the GeneAmp PCR system (SensoQuest GmbH, Göttingen, Germany). The following PCR protocol was used: initial denaturation at 95 ${}^{\circ }$C for 5 min followed by 35 cycles (94 ${}^{\circ }$C for 30 s, 56–66 ${}^{\circ }$C for 30 s and 72 ${}^{\circ }$C for 30 s) and final extension at 72 ${}^{\circ }$C for 5 min. The products were resolved on 6 % denaturing polyacrylamide gel in 1× TAE buffer at 320 V for 3 h (Alaie et al., 2012). Microsatellite alleles were visualized by silver staining (Sanguinetti et al., 1994).

Population genetic indices, namely observed and expected heterozygosity (Levene, 1949), observed and expected number of alleles, Shannon's index and Nei's expected heterozygosity (Nei, 1973) were performed by POPGENE32 software version 1.32 (Yeh et al., 2000). Moreover, polymorphism information content (PIC) was calculated according to Botstein et al. (1980).

## 3 Results and discussion

Microsatellite markers used in this study were previously assessed in different goat breeds (Mei et al., 2006; Marrube et al., 2007; Fatima et al., 2008; Visser and van Marle-Koster, 2009; Dixit et al., 2010; Ramljak et al., 2011). Table 2 shows the measures of genetic diversity at each microsatellite locus in Markhoz goat population. All 10 microsatellite loci were successfully amplified and found to be polymorphic. A total of 52 different alleles were identified with observed allele numbers ranging from 4 to 6 and a mean of 5.2 alleles per locus, being similar to findings obtained by Ganai and Yadav (2001), Gour et al. (2006) and Ramamoorthi et al. (2009) in some Indian goat breeds. The investigated microsatellites showed a good level of polymorphism. The average effective number of alleles was 4.12 varying from 3.46 (BMS1248) to 5.36 (LSCV25). Similar results were found by Dixit et al. (2011) who observed a mean effective number of alleles of 4.2 in Kannai Adu goat; Kumar et al. (2009) who reported a value of 4.7 in Gohilwadi goat and Verma et al. (2010) who found a mean effective allele number of 4.0 in Sangamneri goat breed. Considering that at least four alleles were detected for each microsatellite locus in our population, this is in line with the selective standard of the microsatellite loci given by the Secondary Guidelines for Development of National Farm Animal Genetic Resources Management Plans using reference microsatellite given by FAO (2004) for proficient judgment of genetic distance between breeds. Thus, all studied microsatellite markers exhibited sufficient polymorphism for evaluating intra-population genetic variability in Markhoz goat breed.

**Table 2:** Genetic diversity indices at each microsatellite locus in Markhoz goat.

Locus	Observed	Effective	Observed	Expected	PIC	Shannon's	Nei's
	number of	number of	heterozygosity	heterozygosity		index	expected
	alleles	alleles					heterozygosity
BM2830	6	3.90	1.000	0.748	0.684	1.59	0.73
BM1312	6	5.27	0.827	0.814	0.789	1.71	0.74
LSCV25	6	5.36	0.989	0.817	0.744	1.73	0.62
IDVGA64	5	3.56	1.000	0.723	0.678	1.39	0.72
LSCV06	5	4.20	1.000	0.766	0.793	1.49	0.81
BMS1248	5	3.46	1.000	0.715	0.676	1.35	0.71
IL2RA	5	3.78	1.000	0.749	0.766	1.43	0.77
LSCV36	6	4.43	1.000	0.778	0.691	1.63	0.71
MAP2	4	3.53	1.000	0.721	0.711	1.32	0.72
ILSTS082	4	3.67	1.000	0.731	0.653	1.34	0.81
Mean ± SE	5.2±0.25	4.12±0.22	0.982±0.02	0.756±0.01	0.719±0.02	1.50±0.05	0.73±0.02

PIC represents polymorphism information content.

Heterozygosity is a good measure of the genetic variability within a population. In our sample, the most polymorphic locus was LSCV25 with six alleles and the highest value of expected heterozygosity was 0.817. On the other side, MAP2 was the least polymorphic with four alleles and expected heterozygosity of 0.721. The average expected heterozygosity obtained was 0.756, being lower than the observed one (0.982). This slight excess of heterozygosity was also reported by Ramamoorthi et al. (2009) in Barbari goats. The discrepancy between the observed and expected heterozygosity can be attributed to the random mating among the individuals of the population. However, the available literature generally reports lower values of observed heterozygosity (Kim et al., 2002; Gour et al., 2006; Serrano et al., 2009; Verma et al., 2010). Considering the higher observed heterozigosity in the present population and the lack of allelic fixation at the investigated loci, it is possible to state that Markhoz breed has a wide margin of genetic improvement.

PIC is a parameter indicative of the degree of informativeness of a marker. The PIC value may range from 0 to 1. In the studied population, the average PIC value was 0.719 ranging from 0.653 (ILSTS082) to 0.789 (BM1312). According to the classification of PIC (low polymorphism if PIC value < 0.25, medium if 0.25 < PIC value < 0.50 and high if PIC value > 0.50), this population possessed high genetic diversity. PIC values calculated in the present investigation were comparable with those reported by Ramamoorthi et al. (2009) (0.686) in Barbari goats, Serrano et al. (2009) (0.743) in Spanish Guadarrama breed, Verma et al. (2010) (0.711) in Sangamneri goats, Dixit et al. (2012) (0.770) in Indian goat breeds and by Zhang et al. (2012) (0.781) in Chinese goat breeds. More recently, Radhika et al. (2015) reported an average PIC value of 0.77 in Indian native and crossbred goats, and Wang et al. (2017) stated a mean PIC of 0.716 in Chinese dairy goats. Conversely, lower PIC values were reported in Indian and Pakistani goat breeds (Ganai and Yadav, 2001; Gour et al., 2006; Naqvi et al., 2017), in goats from Korea and China (Kim et al., 2002) and in South African Angora goats (Visser and van Marle-Koster, 2009).

The variability in PIC values found in literature may be due to different mating methods used in the studied populations. In the present study, the high PIC values prove that the microsatellite markers used are highly polymorphic and can be well utilized for molecular characterization of goat germplasm.

Shannon's index measures the biodiversity level in a population (Lewontin, 1972) and it resulted in sufficiently high values for the studied goats with a mean value of 1.50, ranging from 1.32 (MAP2) to 1.73 (LSCV25).

Nei's expected heterozygosity indicates the level of gene diversity within the population (Nei, 1973). In the Markhoz breed it ranged from 0.62 (LSCV25) to 0.81 (LSCV06 and ILSTS082) with a mean value of 0.73. These values were within the range of 0.3–0.8 as determined by Takezaki, and Nei (1996) for markers to be useful to assess genetic variation in a population. Similar results were reported by Rout et al. (2008) in some Indian goat breeds, Dixit et al. (2011) in Kanniadu goats, Verma et al. (2010) in Sangamneri breed and by Rout et al. (2012) in Jamunapari goat.

It is important to underline that it is quite difficult to compare results from other studies as different microsatellite sets have been investigated by different scientists. Thus, comparisons may only give suggestive indication of diversity in a population. Nevertheless, to study the genetic variability in a population plays an important role in developing rational breeding strategies for domestic species.

## 4 Conclusions

Genetic diversity is the main component of the adaptive evolution mechanism because of its preeminent role for the long-term survival probability of all species. Despite the decreasing population size, Markhoz goat genetic diversity seems still conserved. In fact, considering that the sampled population included unrelated animals, the breed seems to have a good level of genetic variability and, as a consequence, a potential margin of adaptability to environment and for future genetic improvement. Nevertheless, further studies should be conducted to deeply evaluate the genetic variability of the Markhoz goat breed.

## Data Availability

The data sets are available upon request from the corresponding author.

## References

[bib1.bib1] Alaie H, Mirhoseini SZ, Mehdizadeh M, Dalirsefat SB (2012). Identification of Complex Vertebral Malformation Carriers in Holstein and Guilan Native Cow Breeds in Iran Using SSCP Markers. Iran, J Appl Anim Sci.

[bib1.bib2] Bahmani HR, Tahmoorespour M, Aslaminiejad AA (2011). Assessment of demographic, geographical and genetic risks in Markhoz Goat population. J Anim Vet Adv.

[bib1.bib3] Bahmani HR, Tahmoorespur M, Aslaminejad AS, Vatankhah M, Rashidi A (2015). Simulating past dynamics and assessing current status of Markhoz goat population on its habitat. Iran, J Appl Anim Sci.

[bib1.bib4] Botstein D, White RL, Skolmick M, Davis RW (1980). Construction of a genetic linkage map in man using restriction fragment length polymorphism. Am J Hum Genet.

[bib1.bib5] Dixit SP, Aggarwal RAK, Verma NK, Vyas MK, Rana J, Sharma A, Chander R (2011). Genetic variability and bottleneck analyses of Kanniadu goat breed based on microsatellite markers. Ind J Anim Sci.

[bib1.bib6] Dixit SP, Verma NK, Aggarwal RAK, Vyas MK, Rana J, Sharma A, Tyagi P, Arya P, Ulmek BR (2010). Genetic diversity and relationship among southern Indian goat breeds based on microsatellite markers. Small Ruminant Res.

[bib1.bib7] Dixit SP, Verma NK, Aggarwal RAK, Vyas MK, Rana J, Sharma A (2012). Genetic diversity and relationship among Indian goat breeds based on microsatellite markers. Small Ruminant Res.

[bib1.bib8] Eriksson G, Namkoong G, Roberds JH (1993). Dynamic gene conservation for uncertain futures. Forest Ecol Manag.

[bib1.bib9] FAO (2004). Secondary guidelines for development of national farm animal genetic resources management plans for global management of cattle genetic resources using reference Microsatellites global projects for the maintenance of domestic
animal genetic diversity (MoDaD).

[bib1.bib10] Farshad A, Akhondzadeh S, Zamiri MJ, Sadeghi GA (2008). The estrous cycle of the Markhoz goat in Iran. Asian-Aust, J Anim Sci.

[bib1.bib11] Fatima S, Bhong CD, Rank DN, Joshi CG (2008). Genetic variability and bottleneck studies in Zalawadi, Gohilwadi and Surti goat breeds of Gujarat (India) using microsatellites. Small Ruminant Res.

[bib1.bib12] Ganai NA, Yadav BR (2001). Genetic variation within and among three Indian breeds of goat using heterologous microsatellite markers. Anim Biotechnol.

[bib1.bib13] Gour DS, Malik G, Ahlawat SPS, Pandey AK, Sharma R, Gupta N, Bisen PS, Kumar D (2006). Analysis of genetic structure of Jamunapari goats by microsatellite markers. Small Ruminant Res.

[bib1.bib14] Groeneveld LF, Lenstra JA, Eding H, Toro MA, Scherf B, Pilling D, Negrini R, Finlay EK, Janlin H, Groenveld E, Weigend S (2010). Genetic diversity in farm animals – a review. Anim Genet.

[bib1.bib15] Kemp SJ, Hishida O, Wambugu J, Rink A, Longeri ML, Ma RZ, Da Y, Lewin HA, Barendse W, Teale AJ (1995). A panel of bovine, ovine and caprine polymorphic microsatellites. Anim Genet.

[bib1.bib16] Kim KS, Yeo JS, Lee JW, Kim JW, Choi CB (2002). Genetic diversity of goats from Korea and China using microsatellite analysis. Asian-Aust J Anim Sci.

[bib1.bib17] Kumar S, Dixit SP, Verma NK, Singh DK, Pande A, Kumar S, Chander R, Singh LB (2009). Genetic diversity analysis of the Gohilwari breed of Indian goat (Capra hircus) using microsatellite markers. Am J Anim Vet Sci.

[bib1.bib18] Levene H (1949). On a matching problem arising in genetics. Ann Math Stat.

[bib1.bib19] Lewontin RC (1972). Testing the theory of natural selection. Nature.

[bib1.bib20] Marrube G, Cano EM, Roldán DL, Bidinost F, Abad M, Allain D, Vaiman D, Taddeo H, Poli MA (2007). QTL affecting conformation traits in Angora goats. Small Ruminant Res.

[bib1.bib21] Mei JIN, Chun-Li G, Jing-Hui HU, Wen-Bo GAO, Wei WANG (2006). Correlation analysis of economic traits in Liaoning new breed of cashmere goats using microsatellite DNA markers. Acta Genet Sinica.

[bib1.bib22] Naqvi AN, Bukhari JF, Vahidi SMF, Utsunomiya YT, Garcia JF, Babar ME, Han J-L, Pichler R, Periasamy K (2017). Microsatellite based genetic diversity and mitochondrial DNA D-Loop variation in economically important goat breeds of Pakistan. Small Ruminant Res.

[bib1.bib23] Nei M (1973). Analysis of gene diversity in subdivided populations. P Natl Acad Sci USA.

[bib1.bib24] Radhika G, Raghavan KC, Aravindakshan TV, Thirupathy V (2015). Genetic diversity and population structure analysis of native and crossbred goat genetic groups of Kerala, India. Small Ruminant Res.

[bib1.bib25] Ramamoorthi J, Thilagam K, Sivaselvam SN, Karthickeyan SMK (2009). Genetic characterization of Barbari goats using microsatellite markers. J Vet Sci.

[bib1.bib26] Ramljak J, Mioč B, Ćurković M, Pavić V, Ivanković A, Međugorac I (2011). Genetic diversity measures of the Croatian spotted goat. Acta Vet.

[bib1.bib27] Rout PK, Joshi MB, Mandal A, Laloë D, Singh L, Thangaraj K (2008). Microsatellite-based phylogeny of Indian domestic goats. BMC Genet.

[bib1.bib28] Rout PK, Thangraj K, Mandal A, Roy R (2012). Genetic Variation and Population Structure in Jamunapari Goats Using Microsatellites, Mitochondrial DNA, and Milk Protein Genes. Sci World J.

[bib1.bib29] Sanguinetti CJ, Neto ED, Simpson AJG (1994). Rapid silver staining and recovery of PCR products separated on polyacrilamide gels. Biotechniques.

[bib1.bib30] Selvaggi M, Tufarelli V, Ventimiglia AM, Birkenhäger JM (2012). Caseins of goat and sheep milk: analytical and technological aspects. Casein: Production, Uses and Health Effects.

[bib1.bib31] Selvaggi M, Laudadio V, Dario C, Tufarelli V (2014). Major proteins in goat milk: an updated overview on genetic variability. Mol Biol Rep.

[bib1.bib32] Selvaggi M, Laudadio V, Dario C, Tufarelli V (2014). Investigating the genetic polymorphism of sheep milk proteins: a useful tool for dairy production. J Sci Food Agr.

[bib1.bib33] Serrano M, Calvo JH, Martínez M, Marcos-Carcavilla A, Cuevas J, González C, Jurado JJ, de Tejada PD (2009). Microsatellite based genetic diversity and population structure of the endangered Spanish Guadarrama goat breed. BMC Genet.

[bib1.bib34] Takezaki N, Nei M (1996). Genetic distances and reconstruction of phylogenetic trees from microsatellite DNA. Genetics.

[bib1.bib35] Verma NK, Dixit SP, Aggarwal RAK, Dangi PS, Joshi BK (2010). Phenotypic and genetic characterization of Sangamneri goat breed. Ind J Anim Sci.

[bib1.bib36] Visser C, van Marle-Koster E (2009). Genetic variation of the reference population for quantitative trait loci research in South African Angora goats. Animal Genetic Resources/Resources Génétiques Animales/Recursos Genéticos Animales.

[bib1.bib37] Wang GZ, Chen SS, Chao TL, Ji ZB, Hou L, Qin ZJ, Wang JM (2017). Analysis of genetic diversity of Chinese dairy goats via microsatellite markers. J Anim Sci.

[bib1.bib38] Yeh FC, Yang R, Boyle TJ, Ye Z, Xiyan JM (2000). POPGENE32, version 1.32. Microsoft Window-based freeware for population genetic analysis.

[bib1.bib39] Zhang L, Yang Q, Li X, Zhou R, Li L (2012). Genetic diversity of five goat breeds in China based on microsatellite markers. Afr J Biotechnol.

